# Supplementary material for: Effects of Preservation and Propagation Methodology on Microcosms Derived from the Oral Microbiome
Source: Microorganisms. 2022 Oct 29;10(11):2146. doi: 10.3390/microorganisms10112146 (PMC9693135; doi:10.3390/microorganisms10112146)
Supplement: Supplementary file 1 [file microorganisms-10-02146-s001.zip › microorganisms-1917463-supplementary.pdf]

Supplementary Information

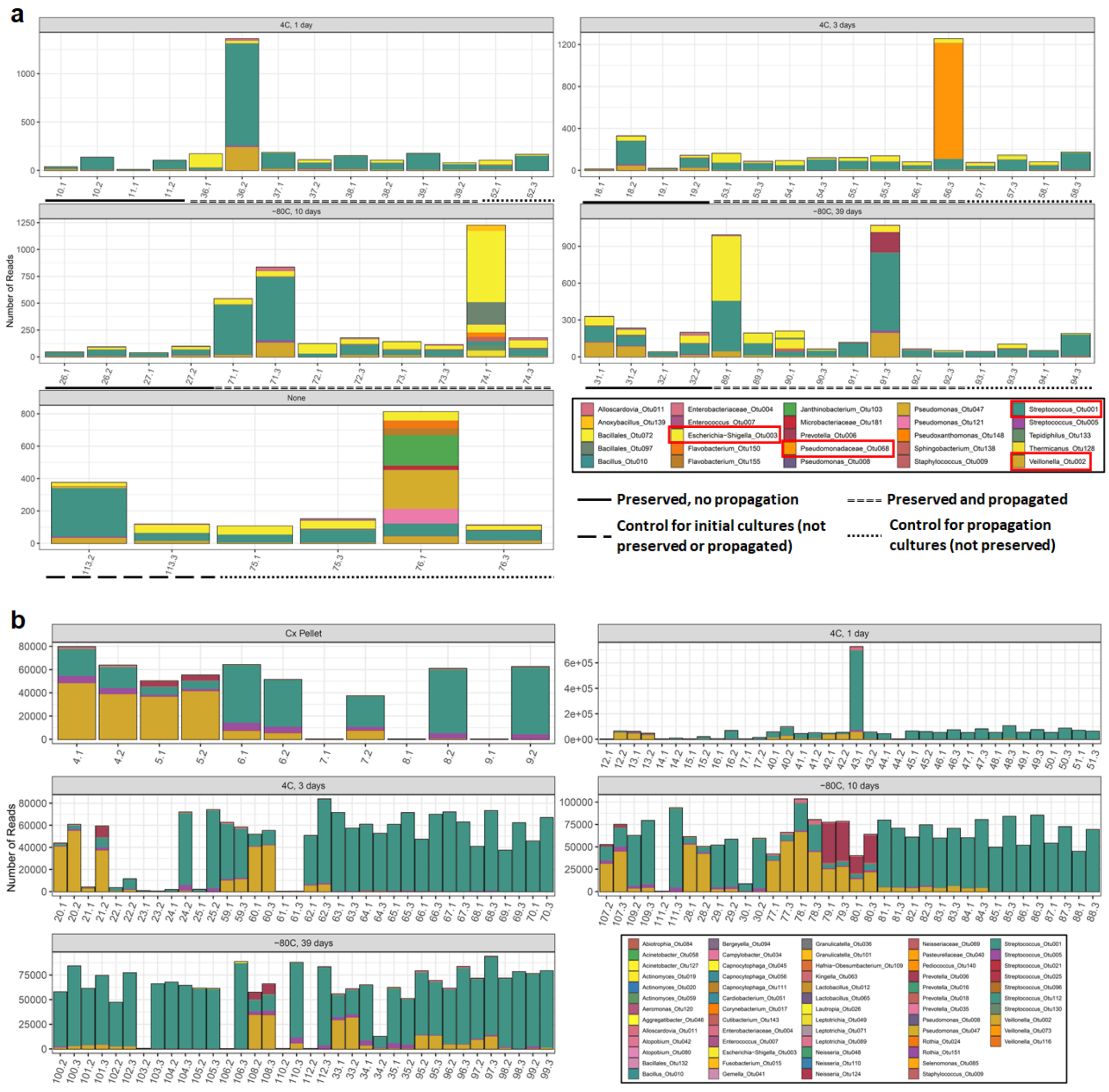

Figure S1. Read counts for preservation experiments for (a) negative controls and (b) sample cultures. Labels in gray boxes indicate preservation conditions. The label of “m.n” is used as follows: ‘m’ index indicates different wells; ‘n’ index indicates different aliquots from the same well. Each control well from the preserved cultures was split into two different wells in the propagation step (Samples 36 and 37 came from Sample 10 and Samples 38 and 39 came from Sample 11). Controls with no preservation condition indicate fresh controls at the time of propagation (Samples 75 and 76 were fresh controls accompanying the preserved and propagated Controls 71 through 74). The presence of Streptococcus OTUs in controls may indicate some cross contamination between cultures and controls. However, read counts in controls were low.

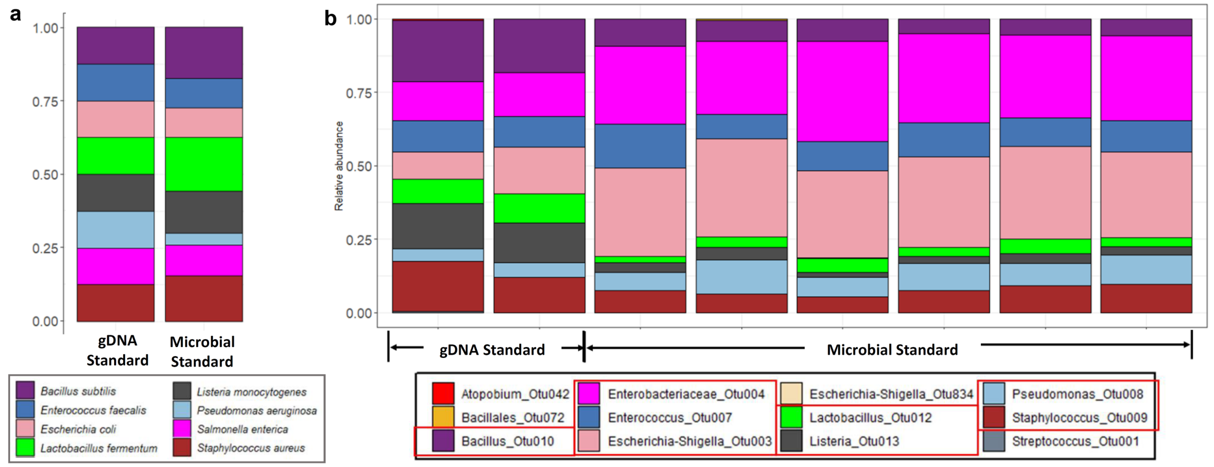

c

Type/Rank	1	2	3	4	5	6	7	8
**Theoretical 16S**	Lactobacillus	Bacillus	Staphylococcus	Listeria	Salmonella	Escherichia	Enterococcus	Pseudomonas
**DNA Standard**	Bacillus	Staphylococcus	Escherichia	Salmonella	Listeria	Lactobacillus	Enterococcus	Pseudomonas
**Microbial Standard**	Escherichia	Salmonella	Enterococcus	Bacillus	Staphylococcus	Pseudomonas	Listeria	Lactobacillus

Figure S2. Analysis of Zymo mock communities. Experimentally, 1μL (10ng) and 200μL ($2.8*10^9$ cells) of the DNA and microbial standards were used, respectively, for a single amplification/extraction sample. (a) Graphical representations of theoretical compositions; (b) Compositions of DNA and microbial standards after rarefaction to 240 reads per sample; (c) Rankings table in order of decreasing relative abundance. "16S only": distribution to be used for 16S rRNA sequencing; "gDNA": distribution to be used for shotgun sequencing. Red boxes in (b) are OTUs expected from the mock communities; red boxes in (c) indicate equivalence between the Salmonella OTU from the theoretical distribution and the Enterobactericeae OTU from the experimental results; green boxes in (c) indicate organisms with similar relative abundances, with the Escherichia, Salmonella, and Listeria OTUs in one group and the Lactobacillus and Enterococcus OTUs in another.

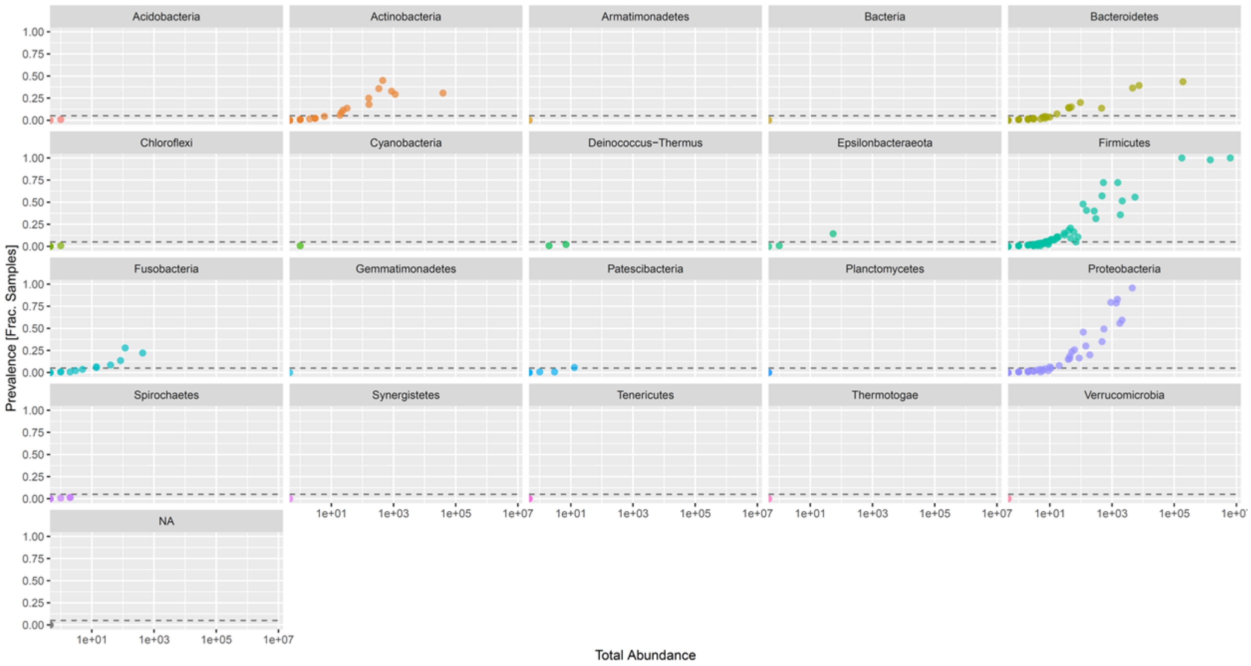

Figure S3. Plots of abundance vs. taxa prevalence for samples with > 1,000 reads. The most prevalent phyla here are Actinobacteria, Bacteroidetes, Firmicutes, Fusobacteria, and Proteobacteria. Other phyla are found in less than 10% of the samples, with cumulative reads fewer than 10,000. NA = unclassified.

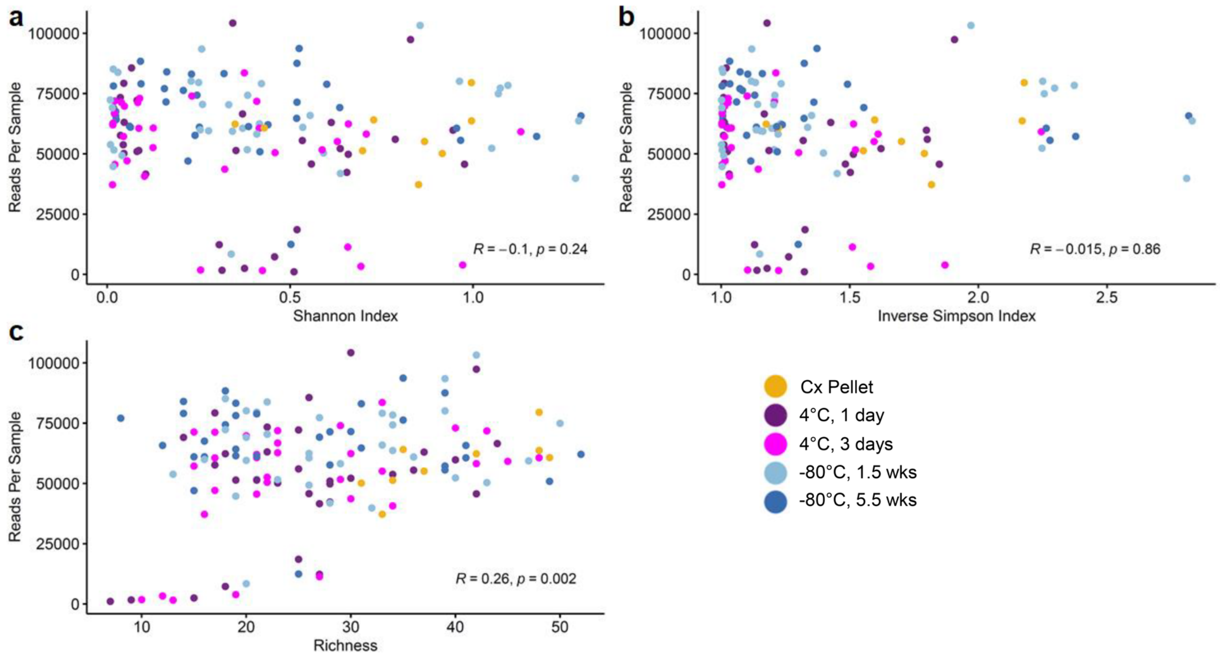

Figure S4. Sequencing depth vs. diversity before rarefaction. Data shown does not include controls, mock communities, an outlier with >700,000 reads, and cultures with <1,000 reads; total number of samples = 144. Shown are (a) Shannon index, (b) inverse Simpson's index, weighted more toward dominant species, and (c) richness.

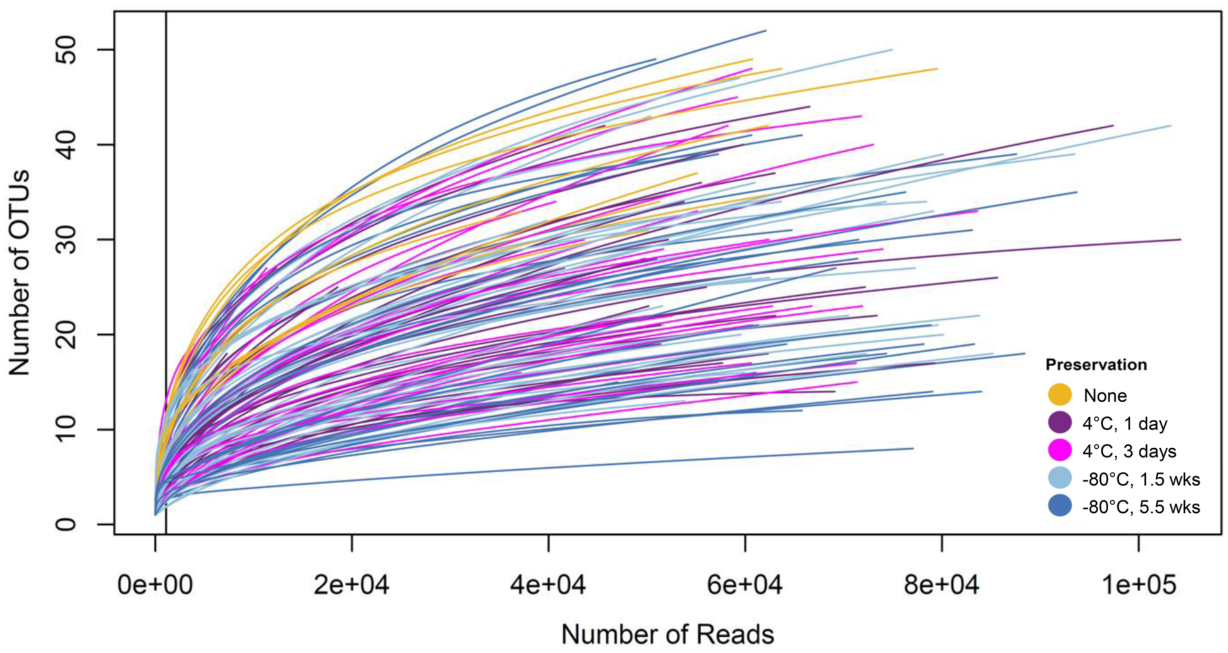

Figure S5. Rarefaction curves. Not shown: controls, original plaque samples, mock communities, and outliers with > 700,000 reads (one sample) or <1000 reads (two samples). Vertical line = 1,082 reads. Cx Pellet = pellet of initial culture (72-hour incubation).

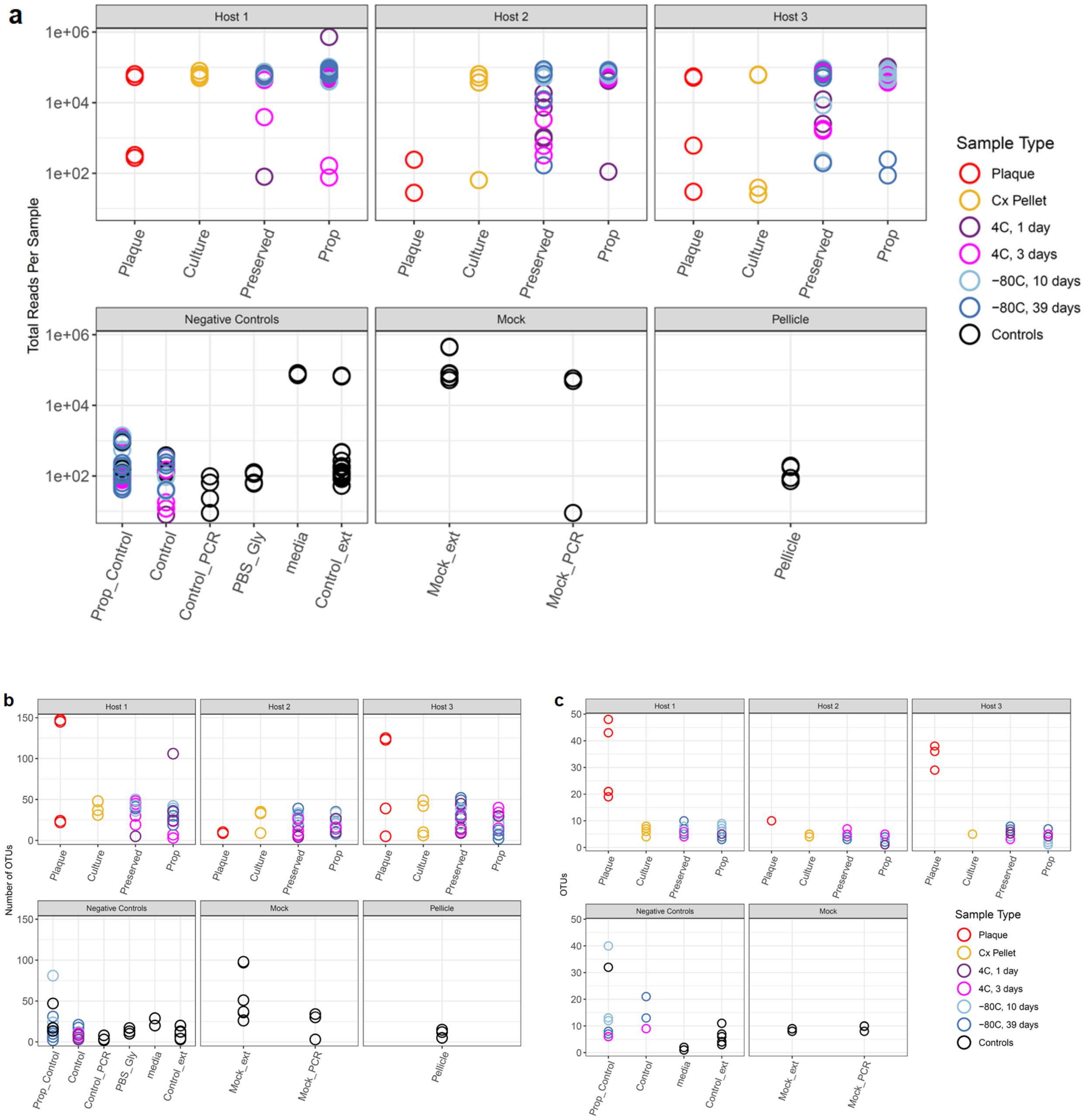

Figure S6. Sequencing depths, sorted by host and sample types. (a) Number of total reads plotted by sample type. PBS/glycerol mix and PCR controls contain approximately 200 reads or fewer. Number of OTUs, before (b) or after (c) rarefaction.

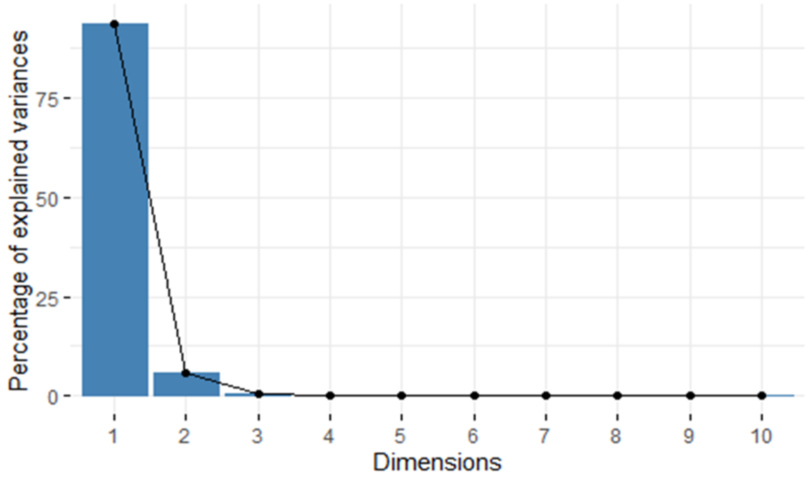

Figure S7. Scree plot of the PCA analysis, showing eigenvalues for principal components.

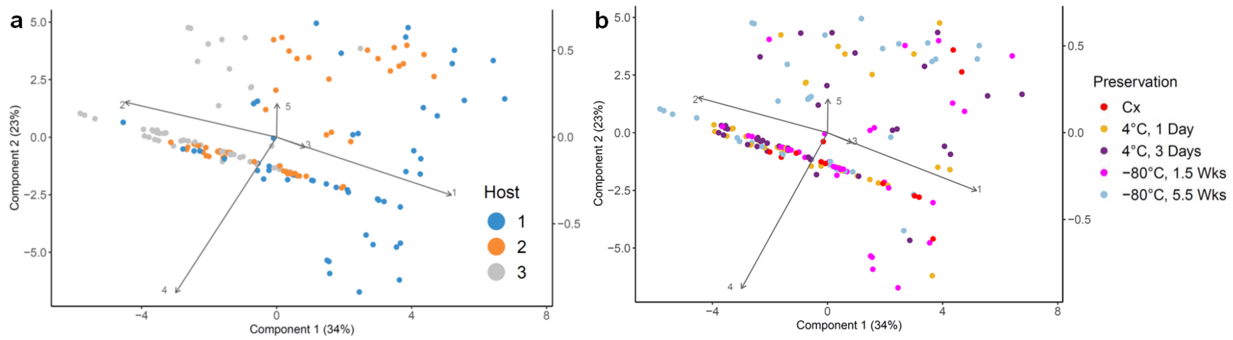

Figure S8. PCA biplots of the isometric log-ratio transforms of non-rarefied relative abundances, (a) by hosts, and (b) by preservation condition.

Supplementary Information: Methods

16S rRNA gene qPCR methods

Quantitative real-time PCR (qPCR) experiments were performed to quantify microorganisms from the in vitro cultures, by comparing DNA samples from these cultures to a standard curve based on *E. coli* strain BL21 (DE3) (New England Biolabs).

Standard Curve: DNA Preparation

To construct the standard curve, we first grew the *E. coli* strain in Luria-Bertani broth (LB). For this step, a sterile loop was used to pick and inoculate a single colony from an existing plate into 10mL of LB broth in a 50mL conical centrifuge tube, which was then incubated at 37°C for 12-16 hours while shaking at 250rpm. After incubation, we performed serial dilutions of the liquid culture down to 10^{-8} and plated 100μL from dilutions between 10^{-5} and 10^{-8} in triplicate on LB agar. These plates were incubated overnight at 37°C, and the resulting colonies were counted the following day to determine the number of colony-forming units (CFU/mL). An important caveat here is that CFU only accounts for viable cells, so it may underestimate the total number of cells and thus be lower than the qPCR copy number.

In addition to counting the CFU/mL, we set aside 1500μL from each dilution and divided this volume equally into three tubes. These samples were centrifuged at 10,000 *x g* to pellet the bacterial cells. Then, each sample was resuspended in 1X PBS, and DNA was extracted with the PowerSoil DNA Isolation Kit (Qiagen 12888) according to the manufacturer's instructions. Concentrations of extracted DNA were measured using the Qubit double-stranded DNA high-sensitivity assay kit (Invitrogen).

Standard Curve: qPCR

qPCR using the extracted DNA was performed using SYBR Green and a 16S rRNA V4 primer set. For the standard curve, a dilution series was established with a starting concentration of 25 ng/μL and subsequently diluted down to 10^{-7} for the PCR reaction. Each dilution was assayed in triplicate in a 96-well plate, with adhesive sealing tape to minimize evaporation. Each PCR reaction was performed in a total volume of 20μL, containing 10μL SYBR Green MasterMix, 1μL

each of forward and reverse primers, 2μL DNA template, and 6μL molecular-grade water. Molecular grade water was also used as the no template control (NTC) and assayed in triplicate. After the reaction mixtures were pipetted into the plate, it was centrifuged at 10,000 *x g* to remove excess bubbles. The qPCR protocol includes an initial denaturation step at 98°C for 3 minutes, an additional 15 seconds of denaturation, and then 30 seconds of annealing at 55°C. This process is repeated for 40 cycles, resulting in a generated melting curve.

Melt curve analysis and agarose gel electrophoresis were used to confirm the expected amplicon. The points for the highest dilutions (10^{-6} and 10^{-7}) were removed from the standard curve to improve the linearity of the curve. The standard curve was run a few times to increase the precision of the efficiencies. Negative controls showed very little DNA, with quantification cycle (Cq) values greater than or equal to 3 cycles (Cq = 33) below that of the lowest concentration (10^{-5} dilution). The r-squared value of the standard curve was 0.99701 and the PCR efficiency calculated from the slope was 82.94%. The standard curve is shown in Figure S9.

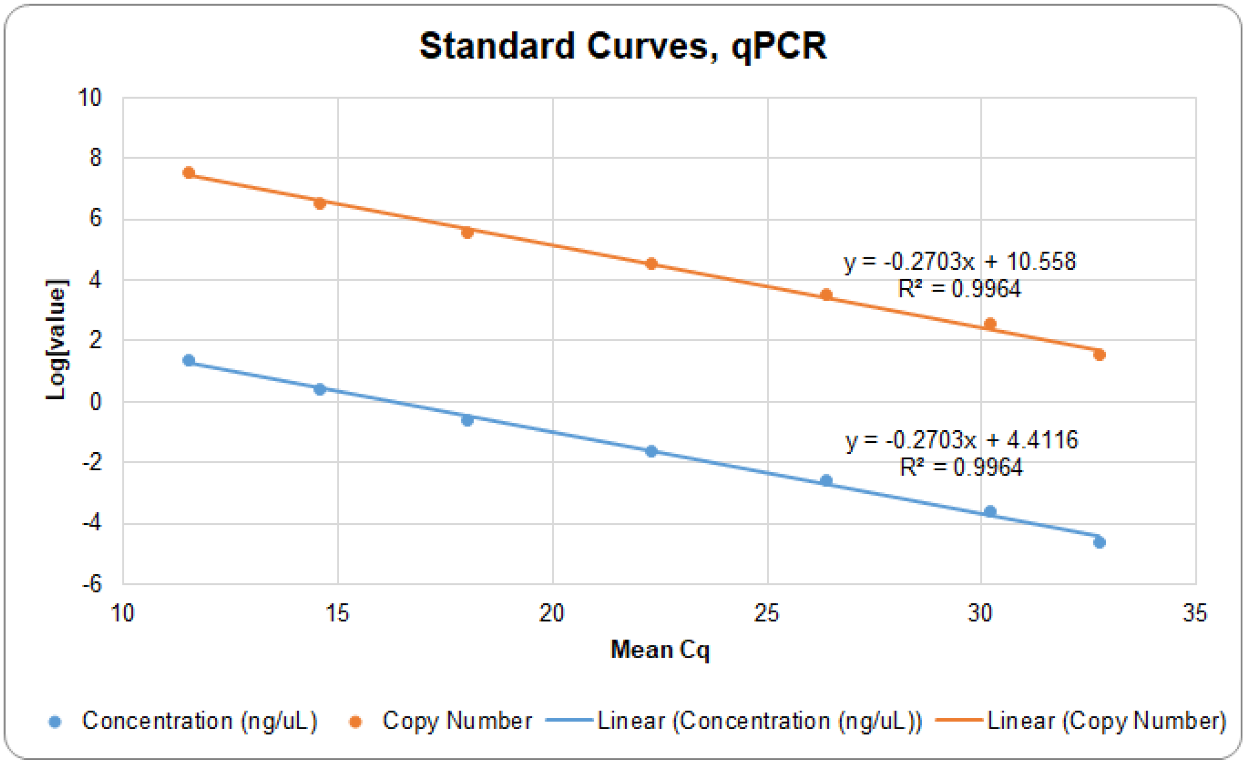

Figure S9. Standard curve of qPCR for 16S rRNA using *E. coli* BL21(DE3) cells.

<u>Extractions from Oral Samples: qPCR</u>

After a working standard curve was established, qPCR was performed on DNA extractions from the oral preservation study using the PowerSoil kit. The *E. coli* standard curve and no-template controls were run on each plate as positive and negative controls, respectively. The entire complement of 142 samples was run in triplicate with a total volume of 20µL per sample. Each sample contained 10µL SYBR Green MasterMix, 1µL each of the V4 forward and reverse primers (Kozich et al., 2013), 4µL DNA template, and 4µL molecular-grade water. Samples that were less than or equal to 3 cycles above the no-template controls were excluded as having insufficient DNA based on the qPCR data, resulting in 33 samples being excluded. Samples with a Cq standard deviation above 1 were rerun. Outlier Cq values were removed from the samples to obtain a more accurate cycle number. The lowest dilution series for each standard curve was $25*10^{-6}$ ng/ul but this final point was removed because the lower limit of detection was at $25*10^{-5}$. Further possible reasons for the consistently low efficiencies include degenerate primers or a long target amplicon length. The values from these samples were thus obtained in duplicate rather than triplicate. Samples EX007 and EX0045 did not amplify and were therefore excluded from the analysis.

<u>Notes</u>

Samples that were ≤3 cycles above the NTCs were removed based on the qPCR data. A total of 33 samples were excluded due to being below the limit of detection. Any samples with a Cq SD above 1 were rerun. Outlier Cq values were removed from the samples in order to obtain a more accurate cycle number. The values from these samples were thus obtained in duplicate rather than triplicate. One of the host samples EX007 and EX0045 did not sample so they were excluded from the analysis.
